# Associations between commute mode and cardiovascular disease, cancer, and all-cause mortality, and cancer incidence, using linked Census data over 25 years in England and Wales: a cohort study

**DOI:** 10.1016/S2542-5196(20)30079-6

**Published:** 2020-05-19

**Authors:** Richard Patterson, Jenna Panter, Eszter P Vamos, Steven Cummins, Christopher Millett, Anthony A Laverty

**Affiliations:** a Public Health Policy Evaluation Unit, School of Public Health, Imperial College London, London, UK; b Medical Research Council Epidemiology Unit, and UK Clinical Research Collaboration Centre for Diet and Activity Research, University of Cambridge, Cambridge, UK; c Population Health Innovation Laboratory, Department of Public Health, Environments and Society, London School of Hygiene & Tropical Medicine, London, UK

## Abstract

**Background:**

Active travel is increasingly recognised as an important source of physical activity. We aimed to describe associations between commute mode and cardiovascular disease, cancer, and all-cause mortality.

**Methods:**

We analysed data from the Office for National Statistics Longitudinal Study of England and Wales (ONS-LS), which linked data from the Census of England and Wales (henceforth referred to as the Census) for 1991, 2001, and 2011 to mortality and cancer registrations. The cohort included individuals traced in the ONS-LS who were economically active (ie, aged ≥16 years, not retired from work, and not a full-time carer). Commuting by private motorised transport, public transport, walking, and cycling were compared in terms of all-cause mortality, cancer mortality, cardiovascular disease mortality, and cancer incidence, using Cox proportional-hazards models with time-varying covariates. Models were adjusted for age, sex, housing tenure, marital status, ethnicity, university education, car access, population density, socioeconomic classification, Carstairs index quintile, long-term illness, and year entered the study, and were additionally stratified by socioeconomic group.

**Findings:**

Between the 1991 Census and the 2011 Census, 784 677 individuals contributed data for at least one Census, of whom 394 746 were included in the ONS-LS and were considered to be economically active working-age individuals. 13 983 people died, 3172 from cardiovascular disease and 6509 from cancer, and there were 20 980 incident cancer cases. In adjusted models, compared with commuting by private motorised vehicle, bicycle commuting was associated with a 20% reduced rate of all-cause mortality (hazard ratio [HR] 0·80, 95% CI 0·73–0·89), a 24% decreased rate of cardiovascular disease mortality (0·76, 0·61–0·93), a 16% lower rate of cancer mortality (0·84, 0·73–0·98), and an 11% reduced rate of incident cancer (0·89, 0·82–0·97). Compared with commuting by private motorised vehicle, rail commuters had a 10% lower rate of all-cause mortality (HR 0·90, 95% CI 0·83–0·97) and a 21% decreased rate of cardiovascular disease mortality (0·79, 0·67–0·94), in addition to a 12% reduced rate of incident cancer (0·88, 0·83–0·94). Walk commuting was associated with 7% lower cancer incidence (HR 0·93, 95% CI 0·89–0·97) Stratified analyses did not indicate differences in associations between socioeconomic groups.

**Interpretation:**

Our findings augment existing evidence for the beneficial health effects of physically active commute modes, particularly cycling and train use, and suggest that all socioeconomic groups could benefit.

**Funding:**

National Institute for Health Research.

## Introduction

The association between commute mode and health can act through several pathways, including physical activity and inhalation of air pollution, with physical activity suggested as the more important of the two.^1^, ^2^ Less well understood pathways include noise and stress. Physical activity improves health in several ways, including reducing all-cause mortality, cardiovascular disease, and some cancers.^3^ In addition to differences in physical activity, car use is also associated with higher ambient amounts of atmospheric pollutants compared with other modes of transport; however, the increased breathing rate of pedestrians and cyclists leads to greater inhaled pollutant doses with these methods of travel.^1^ To investigate the health effect of these exposures, research has been done on links between travel mode and mortality.^4^ For example, analyses of UK Biobank data showed that alternatives to car use were associated with reduced cardiovascular disease mortality and cancer incidence.^5^, ^6^ However, UK Biobank is not nationally representative, with participants being healthier than the general population and, in common with much previous work, the follow-up period is relatively short, with resultant low numbers of events.^5^, ^7^

Although travel by foot, bicycle, and public transport in England and Wales has been declining for four decades, the commute is still a major potential source of physical activity for many working-age people.^8^ In England and Wales, private motorised vehicles are used the most for commuting (67%), followed by public transport (18%), walking (11%), and cycling (3%). However, variations in commute mode are seen, particularly between urban areas (with efficient public transport systems and walkable distances between locations) and rural areas (where residents are more dependent on car use).^8^, ^9^ In England and Wales, commute mode is patterned by socioeconomic groups, with walking and use of public transport more common among more deprived populations and car use more frequent among less deprived populations.^8^ Active travel could, therefore, have the potential to offset well known health inequalities, such as differences in life expectancy and cardiovascular disease rates.^8^, ^10^ However, the association between travel mode and health outcomes across socioeconomic groups is unknown; differences might occur if less affluent populations commute by foot or bicycle because they cannot afford alternatives rather than making a positive choice, which might be more likely among more affluent groups.^8^, ^10^ Findings of a systematic review identified few studies of active commuting and health, scant consideration of cancer outcomes, and very few studies that adequately considered measures of socioeconomic group.^4^ Finally, sample sizes have prevented many previous studies disaggregating commute modes to include cycling and specific public transport modes, despite differences in associated levels of physical activity between public transport modes.^11^

**Research in context**

**Evidence before this study**

Evidence from meta-analyses has shown that bicycle commuting is associated with reduced all-cause mortality, cardiovascular disease incidence, and cancer mortality, whereas walk commuting is associated with decreased cardiovascular disease incidence. However, many studies included in these meta-analyses had a short duration of follow-up and compared people walking or cycling large amounts during commutes with those accumulating shorter amounts. These studies did not make comparisons with people who commuted using private motorised transport. Primary studies comparing car commuters with other modes of commuting have found that cycling was associated with reduced all-cause mortality, cardiovascular disease, and cancer incidence and mortality, whereas walk commuting was associated with reduced cardiovascular disease. A systematic review of the health benefits of public transport use found that initiating public transport was associated with reduced adiposity, but few cohort studies have investigated non-adiposity outcomes. Potential mechanisms for these associations include physical activity, atmospheric pollution, and wellbeing.

**Added value of this study**

Our study draws on a nationally representative data source (the Census of England and Wales), with individuals contributing up to 25 years of follow-up. The large dataset uniquely allows a longer term investigation of public transport users stratified by train and bus use; it also allows investigation of differences across socioeconomic groups not previously studied. Cycling to work was associated with reduced all-cause mortality, cardiovascular disease mortality, cancer mortality, and cancer incidence. Rail commuters had decreased all-cause mortality and cardiovascular disease mortality in addition to incident cancer. Walk commuting was associated with a reduced incidence of cancer. Stratified analyses did not show any differences in associations between socioeconomic groups.

**Implications of all the available evidence**

Physically active commute modes, particularly cycling and train use, are associated with a range of health benefits compared with car use. Policy makers in the health sector and beyond should consider the health effects of decisions that affect people's travel choices.

We aimed to extend previous research using data from a population-based linkage study over a longer period than used in many previous studies to investigate the effects of commute mode on cardiovascular disease mortality, cancer mortality, all-cause mortality, and incident cancer. The large sample size and long follow-up period enabled assessment of potentially differential effects across socioeconomic groups.

## Methods

### Sample

We obtained data from the UK Office for National Statistics Longitudinal Study of England and Wales (ONS-LS), a dataset that links data from several sources including the Census of England and Wales (henceforth referred to as the Census) and registrations of death and cancer diagnoses. Participant tracing and data linkage are coordinated by the UK Office for National Statistics (ONS) to enable social and demographic research. Participants contributed complete data for at least one eligible Census (1991, 2001, and 2011) and were followed up until the end of 2016 (2015 for cancer incidence). Census participation is a legal requirement in the UK, leading to high response rates (eg, 94% in 2011).^12^ The ONS-LS samples approximately 1% of the population from each Census, based on four undisclosed birth dates in the calendar year (four of 365) and reports high linkage rates (98·8% of eligible individuals in 2011) allowing data from consecutive Censuses to be linked together, in addition to enabling the linkage of events data, such as mortality and cancer registrations.^13^ For example, ONS-LS personnel linked individuals to the National Cancer Registration and Analysis Service (England) and the Welsh Cancer Intelligence and Surveillance Unit (Wales), which are systematic collections of data for instances of cancer.^14^ In addition to individual data for exposure and confounders from the Census, the ONS-LS also contains variables derived from Census data about participant's neighbourhood of residence, including population density and area-based measures of socioeconomic group. To analyse commute mode, we restricted the sample to economically active people (ie, excluding children <16 years, full-time carers, and people who were retired from work).

### Variables

The primary exposure was usual commute mode, which was divided into four categories: private motorised mode (eg, car or motorbike), public transport (eg, bus or rail), walking, or cycling. People working from home were excluded. To investigate associations between health outcomes and different forms of public transport individually, we did analyses disaggregated into bus and rail. Exposure data were derived from responses to the Census question about usual commute mode, for which individuals selected one from a list of travel modes. The travel modes listed varied slightly from Census to Census, with a full list of modes available in each Census provided in the appendix (p 3).

We assessed four outcomes: all-cause mortality (assessed by death registrations), cardiovascular disease mortality (defined as deaths classified by International Classification of Diseases tenth revision [ICD-10] codes I20–25 and I60–69), cancer mortality (defined as ICD-10 codes C00–C97 and D37–84), and incident cancer (assessed by cancer registrations). For deaths classified under the previous International Classification of Diseases ninth revision (ICD-9) coding system, equivalent codes were used (appendix p 3). Dates of death and cancer registrations were provided by the ONS-LS to the nearest month; a more specific time of death was not available because of the risk of disclosure of identity. Analyses only considered a participant's first incidence of cancer registration, because subsequent diagnoses might be recurrences, metastases, or another primary cancer, which are likely to have differing aetiological pathways.^15^

We regarded measures of participant demographics as potentially confounding variables: age, age^2^, sex, and ethnicity (minority ethnic group or white). We also assessed household circumstances using housing tenure (homeowner or non-homeowner), marital status (married or non-married), and presence of a long-term illness (yes or no). We included workplace and socioeconomic variables: university education (no degree or has a degree), ONS socioeconomic classification of occupation (NSSEC; managerial or higher, intermediate, or routine or manual), and individual-level quintile of their ward's Carstairs index (a composite of male unemployment, lack of car ownership, overcrowding, and social class of household head).^16^, ^17^, ^18^, ^19^, ^20^, ^21^ A ward is a small unit of geography in England and Wales with a mean population of 6600. We judged access to a car (yes or no) as a potential determinant of car use and population density of the participant's ward of residence (<2000 people per km^2^ or ≥2000 people per km^2^) as a measure of neighbourhood built environment. Finally, we included year of cohort entry in analyses, to account for changes over time. Neighbourhood measures (population density and Carstairs index) were based on the individuals' ward of residence. The categorisation of ethnicity, education, and marital status was limited by available data and comparability of variables over time, whereas population density was available as a categorical variable dichotomised as close as possible to the median in the absence of a strongly evidence-based alternative.

### Statistical analysis

We summarised characteristics of individuals by usual commute mode. For people who contributed data from two or more Censuses, baseline data reflect information provided in the earliest included Census.

We followed up individuals from the date of their first eligible Census until either they were no longer eligible (eg, not employed at a subsequent Census), they were absent from a subsequent Census, their date of death was registered, their emigration was registered, or until the end of the study period (December, 2016, for mortality or December, 2015, for cancer incidence analyses). For cause-specific mortality analyses, we additionally censored individuals at the date of death from other causes. In the analyses of incident cancer, we excluded people with a history of cancer and censored individuals at death.

We used Cox proportional-hazards regression models to estimate hazard ratios (HRs) with 95% CIs for the associations between commute mode and the study outcomes.^22^, ^23^ Models were adjusted for age, sex, housing tenure, marital status, ethnicity, university education, car access, population density, NSSEC, Carstairs index quintile, long-term illness, and year entered study. Commute mode was entered as a time-varying exposure in the models, which allowed individuals who reported different modes in different Censuses to contribute data to all commute modes they reported. All confounding variables were judged time-varying covariates, except for age, sex, ethnicity, limiting long-term illness, and year of study entry, which were sourced from baseline and were regarded as time invariant for these analyses. For people contributing data to multiple Censuses, we allowed socioeconomic, household, and workplace characteristics to vary over each 10-year period and entered these as time-varying covariates in the models. We evaluated the proportional-hazards assumption by plotting Schoenfeld residuals against survival time and by regressing the scaled Schoenfeld residuals on functions of time to test for a non-zero slope.

We used a four-category measure of commute mode (private motorised vehicle, public transport, walk, and bicycle) in analyses of outcomes (all-cause mortality, cardiovascular disease mortality, cancer mortality, and cancer incidence); we also disaggregated public transport users into bus and rail users to investigate any differences between these two modes of transport. We tested potentially modifying factors for an interaction and, when appropriate, we stratified analyses to look at potentially differential effects across groups. Stratification factors were the three categories of NSSEC (managerial or higher *vs* intermediate *vs* routine or manual), population density (<2000 people per km^2^ *vs* ≥2000 people per km^2^), full-time working versus part-time working (only available in 2001 and 2011 Censuses), and sex (male *vs* female). To assess for a potential dose-response, we used the subset of data from 2001 and 2011 Censuses, which contained straight-line commute distance. Commuters were dichotomised into short-distance and long-distance commuters, based on being higher or lower than mode-specific median commute distances. All analyses used private motorised transport commuters as the reference category.

To investigate the effect of changing commute mode, we compared people who continued using private motorised vehicles for commuting in 1991 and 2001 Censuses with those who switched from a private motorised vehicle in 1991 to physically active commuting in 2001; we also compared people who continued physically active commuting in 1991 and 2001 Censuses with those who changed from active modes in 1991 to commuting by private motorised vehicle in 2001. Participants were followed up from 2001 to 2016 (2015 for incident cancer analyses).

Sensitivity analyses added both self-reported health and presence of a limiting illness as covariates, which were first added to the Census in 2001. We also investigated potential reverse causality, with analyses that excluded the first 2 years of follow-up subsequent to each Census (eg, to minimise the effect of people with pre-existing poorer health selecting less active commute modes). All analyses were done with Stata version 15.

### Role of the funding source

The funder had no role in study design, data collection, data analysis, data interpretation, or writing of the report. The corresponding author had full access to all data in the study and had final responsibility for the decision to submit for publication.

## Results

Between the 1991 Census and the 2011 Census, 784 677 individuals contributed data for at least one Census and were traced in the ONS-LS. Of these individuals, 416 871 were economically active (ie, aged ≥16 years, not retired from work, and not a full-time carer; appendix p 4), of whom 15 394 reported either working from home or their commute mode as other and were excluded. Additional exclusions were 5038 people with missing data for commute mode and 1693 with missing data for at least one covariate. In total, 394 746 economically active working-age individuals were available for analysis, although samples differed slightly between analyses. Comparison of people excluded because of missing data and those who were included shows that people excluded were least likely to use private motorised vehicle for commuting (53·1% *vs* 65·5%) and to have a university degree (7·5% *vs* 20·6%) and were most likely to be aged 60 years or older (15·4% *vs* 4·2%; appendix p 5). During the study period (from April, 1991, to December, 2016 [for mortality], or December, 2015 [for cancer incidence]), 13 983 people died, 3172 from cardiovascular disease and 6509 from cancer, and there were 20 980 incident cancer cases.

At baseline, 65·5% of individuals used a private motor vehicle for commuting, 18·8% used public transport, 12·4% walked, and 3·2% cycled (table). Participants aged 30–59 years were most likely to be private motor vehicle commuters compared with people in other age groups; for example, 68·0% of individuals aged 30–44 years were private motor vehicle commuters compared with 61·9% of those aged 16–29 years. People aged 16–29 years were most likely to use public transport for commuting (22·5%), whereas those aged 45–59 years were least likely to use public transport for their commute (15·7%). Men were more likely than women to commute by private motor vehicle (71·6% *vs* 58·7%) or bicycle (4·0% *vs* 2·3%) but were less likely to use public transport for their commute (15·6% *vs* 22·5%) or to walk (8·7% *vs* 16·6%). Participants of white ethnicity were more likely than minority ethnic groups to be private motor vehicle commuters (67·2% *vs* 50·9%) or bicycle commuters (3·4% *vs* 1·4%) and were less likely to be public transport commuters (16·9% *vs* 35·2%). People living in areas with fewer than 2000 people per km^2^ were more likely to be private motor vehicle commuters than were those living in areas with a population density of at least 2000 people per km^2^ (75·3% *vs* 57·9%) and were less likely to use public transport for their commute (10·6% *vs* 25·2%). Characteristics of people using different commute modes at the 1991, 2001, and 2011 Census are provided in the appendix (pp 6–8); disaggregated public transport use is also shown in the appendix (p 9). Although some changes over time are seen (eg, the proportion of people with a university degree), the relations between variables and commute mode remain broadly similar.

**Table:** Baseline characteristics of individuals and commute mode, 1991–2016

		**Total (n)**	**Private motorised (%)**	**Public transport (%)**	**Walk (%)**	**Bicycle (%)**
All		394 746	65·5%	18·8%	12·4%	3·2%
Age, years						
	16–29	149 593	61·9%	22·5%	12·3%	3·2%
	30–44	142 958	68·0%	17·1%	11·6%	3·3%
	45–59	85 674	67·8%	15·7%	13·4%	3·0%
	≥60	16 521	64·3%	16·8%	15·7%	3·2%
Sex						
	Male	209 510	71·6%	15·6%	8·7%	4·0%
	Female	185 236	58·7%	22·5%	16·6%	2·3%
Housing tenure						
	Homeowner	288 584	71·3%	15·8%	10·0%	2·9%
	Non-homeowner	106 162	49·8%	27·1%	18·9%	4·2%
Marital status						
	Unmarried	190 566	60·0%	23·6%	13·0%	3·4%
	Married	204 180	70·7%	14·4%	11·9%	3·0%
Ethnicity						
	White	353 842	67·2%	16·9%	12·4%	3·4%
	Minority ethnicity	40 904	50·9%	35·2%	12·5%	1·4%
University education						
	No degree	313 292	66·3%	17·1%	13·4%	3·2%
	Has a degree	81 454	62·5%	25·4%	8·7%	3·4%
Car access						
	No car access	53 952	17·9%	47·6%	27·9%	6·7%
	Has access to a car	340 794	73·1%	14·3%	10·0%	2·7%
Population density, people per km^2^						
	0–2000	172 330	75·3%	10·6%	11·2%	2·9%
	≥2000	222 416	57·9%	25·2%	13·4%	3·5%
NSSEC—social class						
	Managerial or higher	130 264	70·4%	19·7%	7·3%	2·6%
	Intermediate	98 298	69·4%	19·1%	9·6%	1·9%
	Routine or manual	166 184	59·4%	18·0%	18·1%	4·5%
Carstairs index quintile						
	1 (least deprived)	76 946	78·1%	11·0%	8·0%	2·8%
	2	79 574	72·4%	13·9%	10·6%	3·1%
	3	80 434	66·7%	16·6%	13·2%	3·5%
	4	81 109	61·0%	20·9%	14·6%	3·4%
	5 (most deprived)	76 683	49·3%	32·0%	15·6%	3·2%
Long-term illness						
	No illness	376 939	65·6%	18·8%	12·3%	3·2%
	Has an illness	17 807	63·1%	19·9%	14·3%	2·7%
Entered study						
	1991	209 217	68·4%	16·3%	12·0%	3·3%
	2001	88 633	65·2%	19·3%	12·4%	3·1%
	2011	96 896	59·6%	23·9%	13·3%	3·1%
Events						
	All-cause mortality	13 983	67·8%	15·9%	13·1%	3·1%
	Cardiovascular disease mortality	3172	69·9%	15·1%	12·1%	2·9%
	Cancer incidence	20 980	73·0%	12·9%	11·4%	2·7%
	Cancer mortality	6509	64·4%	18·2%	14·0%	3·5%

Data obtained from the Office for National Statistics Longitudinal Study of England and Wales. NSSEC=Office for National Statistics socioeconomic classification of occupation.

**Figure 1:**
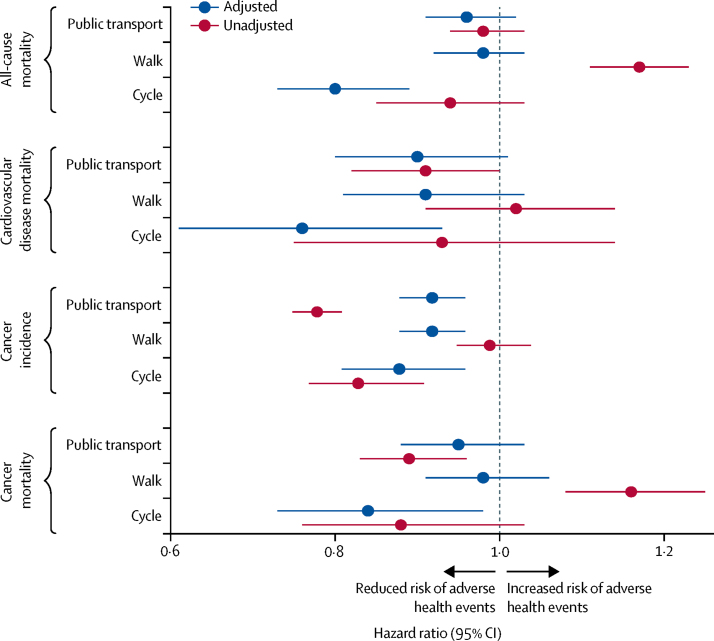
Mortality and cancer outcomes by commute mode, 1991–2016 Data obtained from the Office for National Statistics Longitudinal Study of England and Wales. Number of events and adjusted and unadjusted hazard ratios (95% CIs) are in the appendix (p 10). Hazard ratios are for usual commute modes compared with a reference category of private motorised vehicle commuting. Model adjusted for age, sex, housing tenure, marital status, ethnicity, university education, car access, population density, Office for National Statistics socioeconomic classification of occupation, Carstairs index quintile, long-term illness, and year entered study.

Compared with people who commuted by private motorised vehicle, bicycle commuters had a 20% reduced rate of all-cause mortality (HR 0·80, 95% CI 0·73–0·89), a 24% reduced rate of cardiovascular mortality (0·76, 0·61–0·93), an 11% reduced rate of incident cancer (0·89, 0·82–0·97), and a 16% reduced rate of cancer mortality (0·84, 0·73–0·98) in adjusted models (figure 1; appendix p 10). Walk commuters had a 7% lower rate of incident cancer (HR 0·93, 0·89–0·97) in adjusted models, and in unadjusted analyses, walk commuters had 17% and 16% higher rates of all-cause mortality (HR 1·17, 95% CI 1·11–1·23) and cancer mortality (1·16, 1·08–1·25), respectively. Public transport commuters had a 7% lower rate of incident cancer compared with commuters using a private motorised vehicle (HR 0·93, 95% CI 0·89–0·97) in adjusted models, with larger associations in unadjusted analyses (0·79, 0·76–0·82).

**Figure 2:**
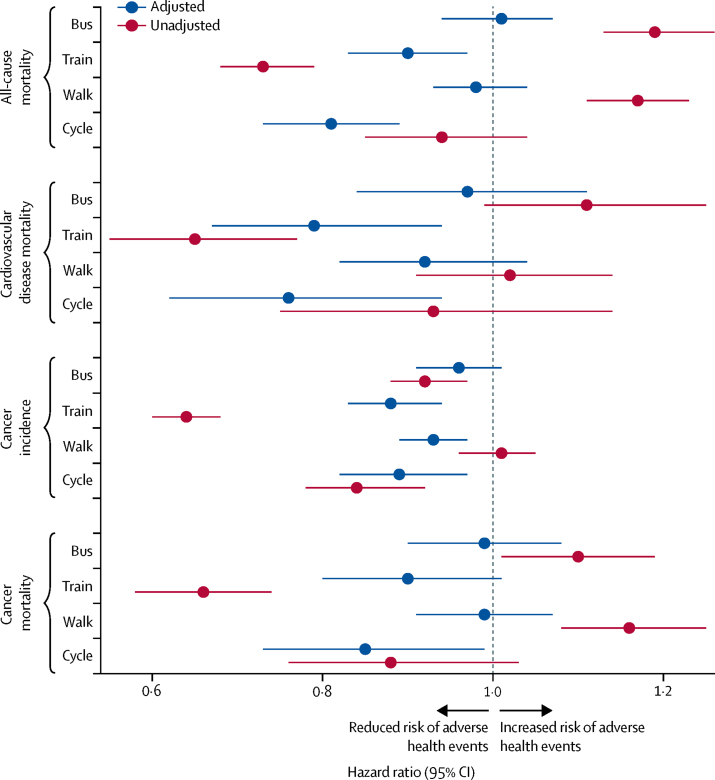
Mortality and cancer outcomes by commute mode, 1991–2016, disaggregated by public transport users Data obtained from the Office for National Statistics Longitudinal Study of England and Wales. Number of events and adjusted and unadjusted hazard ratios (95% CIs) are in the appendix (p 10). Hazard ratios are for usual commute modes compared with a reference category of private motorised vehicle commuting. Model adjusted for age, sex, housing tenure, marital status, ethnicity, university education, car access, population density, Office for National Statistics socioeconomic classification of occupation, Carstairs index quintile, long-term illness, and year entered study.

Analyses disaggregating public transport showed that rail commuters had a 10% lower rate of all-cause mortality (HR 0·90, 95% CI 0·83–0·97), a 21% lower rate of cardiovascular disease mortality (0·79, 0·67–0·94), and a 12% lower rate of incident cancer (0·88, 0·83–0·94) compared with private motorised vehicle commuters, whereas bus commuters showed no differences (figure 2; appendix p 10). In unadjusted analyses, bus commuters had poorer outcomes than did rail commuters; for example, bus commuters had a 19% higher rate of all-cause mortality (unadjusted HR 1·19, 95% CI 1·13–1·26) whereas rail commuters had a 27% lower rate of all-cause mortality (0·73, 0·68–0·79).

**Figure 3:**
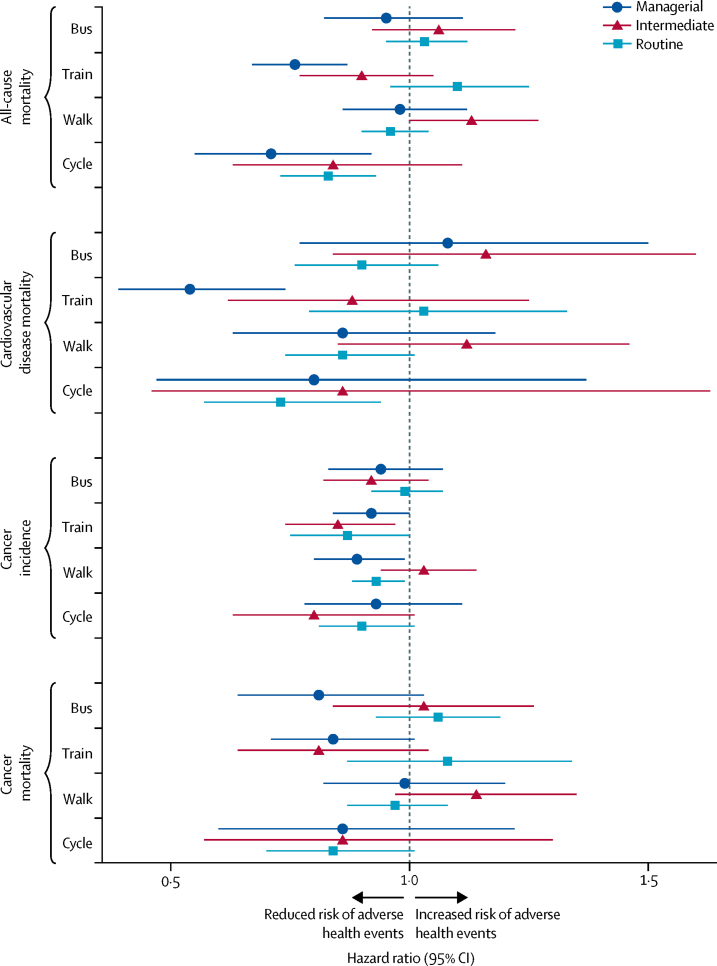
Mortality and cancer outcomes by commute mode, 1991–2016, stratified by Office for National Statistics socioeconomic classification of occupation Data obtained from the Office for National Statistics Longitudinal Study of England and Wales. Number of events and adjusted and unadjusted hazard ratios (95% CIs) are in the appendix (p 12). Hazard ratios are for usual commute modes compared with a reference category of private motorised vehicle commuting. Model adjusted for age, sex, housing tenure, marital status, ethnicity, university education, car access, population density, Office for National Statistics socioeconomic classification of occupation, Carstairs index quintile, long-term illness, and year entered study.

Tests for interaction showed that population density and part-time working had no effect on associations (appendix p 11). Analyses stratified by social classification suggested that associations were of similar magnitude and direction across occupation-based socioeconomic group (figure 3; appendix p 12). In sex-stratified analyses, no differences were seen between men and women (appendix p 13).

Median commute distances varied from 0·8 km for walk commuters to 13·1 km for rail commuters (appendix p 14). Comparison of outcomes between commuters above and below the median distance for their commute mode was inconclusive (appendix p 15). Analyses of changing commute mode were consistent with no effect (appendix pp 16–18).

Sensitivity analyses additionally adjusting for self-reported health produced similar results as did those excluding the first 2 years of follow-up subsequent to each Census (appendix pp 19–20).

## Discussion

The findings of this analysis of data from ONS-LS showed that bicycle commuting was associated with a reduced rate of all-cause mortality, cardiovascular disease mortality, and cancer mortality, and incident cancer, compared with commuting by private motorised vehicle. Rail commuting was associated with a decreased rate of all-cause mortality and cardiovascular disease mortality, and incident cancer, whereas walk commuting was associated with a reduced rate of incident cancer. These associations were found to be similar across socioeconomic groups.

These analyses make use of large representative population-based data with high levels of linkage to mortality and cancer data. Individuals contributing up to 25 years of follow-up, and the large sample size, allowed granularity in assessing commute modes, including specific analyses of cycling and separating public transport into bus and rail. Nonetheless, this large and long-term dataset has some limitations because of the lack of granularity in some variables of interest and the absence of others. First, the use of usual commute mode as the exposure is a simplification and fails to capture travel for other reasons, use of different modes during the same day, use of different modes on different days, and people who only commute on specific days (including part-time working). This limitation could drive some of the differences noted between these findings and those of researchers using more detailed measures of commute mode. Second, assessments of commute mode were made 10 years apart, and if individuals changed their commute mode it is uncertain exactly when that happened, which introduces some measurement error. Comparing commute mode in consecutive Censuses showed that people who changed mode were more likely to change to private motorised vehicle than any other mode, the effect of which is likely to bias towards the null (appendix p 21). Third, we did not have data for some potentially important confounding variables, such as air quality, dietary intake, adiposity, smoking, non-commuting physical activity, drugs, and comorbidities. The potential effects of some of these missing variables are uncertain because there are conflicting findings on the association of active commuting with leisure time physical activity and on the effects of adiposity on transport mode choice.^24^, ^25^ Additionally, adiposity is potentially on the causal pathway between commute mode and our outcomes, so although it would have been good to assess the importance of this factor, its effects on our results remain uncertain. However, we did adjust for a range of covariates and did sensitivity analyses, which included self-reported health and excluded the first 2 years of follow-up, and these analyses produced similar findings to our main findings. Nonetheless, it is plausible that more detailed medical history and lifestyle data would have allowed for more complete adjustment for potentially confounding factors.

We were unable to investigate the direct or indirect mechanisms behind our findings. Studies comparing the relative importance of physical activity and atmospheric pollutants in the health effects of transport suggest that physical activity is likely to predominate.^1^, ^2^ Other mechanisms, such as stress during journeys, might also account for some of our findings, although these mechanisms are complex and context dependant.^26^ Our results represent the health associations of using different commute modes and are likely to encompass multiple pathways. Further research is needed to better elucidate the mechanisms that might predominate and in which contexts. Many of our supplemental analyses had wide CIs, leading to inconclusive findings. A low number of events in subgroups might also have led to our inability to detect interactions with postulated modifiers (eg, population density and part-time working). Other potential causes of the inconclusive findings include the measures available in the data; for example, use of straight-line commute distance, which is likely to be an imprecise proxy for commute dose, and use of population density as a measure of the built environment. Measurement error associated with only having commute mode recorded every 10 years possibly contributed to the paucity of findings in the analyses of changing mode.

Our results broadly concur with other research on the health effects of travel modes, although differing follow-up times and exposure classifications make exact comparisons difficult. We identified smaller associations for walk and bicycle commuting than did a systematic review and meta-analysis from 2018.^4^ For example, the systematic review found that bicycle commuting was associated with rate reductions of 24% for all-cause mortality and 25% for cancer mortality, compared with 20% and 16%, respectively, in this analysis. These differences could be attributable to the systematic review comparing the most active commuters with the least active, which is likely to yield larger differences than the analyses in this study, which were not restricted to commuters on these extremes.^4^, ^27^, ^28^ A separate systematic review that only investigated bicycle commuting found that this mode was associated with reduced rates of incident and fatal cancer, although meta-analyses were not done.^29^

Associations identified in a 2018 study using UK Biobank data with 5 years of follow-up were also larger than those reported in our study.^5^ For example, rate reductions for bicycle commuting and all-cause mortality and cardiovascular disease mortality were 41% and 52%, respectively, compared with 20% and 24% in our study. Another study using UK Biobank data investigated commuting and non-commuting travel with modes dichotomised into people who travelled exclusively by car and those who used any other mode or combination of modes.^6^ Those findings were broadly consistent with ours, in that more physically active travel modes were associated with lower all-cause mortality.^6^ Differences between the findings of UK Biobank and our analysis could be attributable to variations in exposures, with UK Biobank participants being able to select multiple travel modes; a divergence in sample demographic characteristics, with UK Biobank recruited from volunteers and only those aged 40 years or older; and discrepancies in available covariates, with UK Biobank gathering detailed data for comorbidities, diet, non-commuting physical activity, and smoking. Importantly, the UK Biobank sample, although large, is not representative of the UK population, unlike our sample, which increases the potential generalisability of our results.^7^

The health effects of public transport use are less well understood than are those of walking and cycling. However, public transport users accumulate physical activity in the course of their public transport journeys, typically by walking to connect journeys from their origins or to their ultimate destinations.^11^, ^30^, ^31^ A systematic review of longitudinal studies additionally found that initiating public transport use was associated with an 0·30 kg/m^2^ reduction in body-mass index, although there remains a paucity of high-quality studies in this area.^32^ However, our study is one of the first to examine whether public transport use is associated with incident mortality. The positive associations seen were primarily among rail commuters rather than bus commuters. This finding could be attributable to rail users accumulating more physical activity in the course of their journeys than bus users, which is consistent with bus stops being more densely located than train stations and, therefore, more likely to be closer to journeys' origins or ultimate destinations.^11^, ^33^ Other possible explanations are differences in reliability, speed, and levels of crowding, which can affect stress levels of those using buses and trains differently. Evidence also suggests that bus users have greater exposure to atmospheric pollutants than do rail commuters.^1^ Train commutes are, on average, longer than bus commutes, thus some of the differences might be attributable to people taking on longer (train) commutes to get access to an increased salary, better-quality housing, an upgraded neighbourhood of residence, or a combination of these, which themselves might lead to improved outcomes.^26^ Train users are also likely to be more socioeconomically advantaged than are bus users, in this sample and elsewhere,^33^ meaning that residual confounding could account for some differences between these modes. The NSSEC stratified results provide no evidence for differences across socioeconomic groups, which accords with other research and indicates that potential health benefits would be similar across socioeconomic groups.^6^ Alternatively, other notions of socioeconomic group (eg, education, absolute income, or wealth) might find differing results, or the relatively low numbers of events in these stratified analyses could have led to a lack of power to detect differences.

The mixed findings for analyses of commute distance might be because of low statistical precision, because commute distance was not available for all individuals. Straight-line distance between residential and workplace addresses is unlikely to be an accurate measure of commute distance, and the degree to which this imprecision is the case could vary between modes; for example, it seems plausible that walking is more closely aligned with straight-line distance than is train travel. Commute time data were not available, but these data might provide a more valid measure of exposure to relevant pathways across modes, in particular for effects through a wellbeing mechanism as a result of long or stressful commutes. The suggestive finding that longer distance commutes were associated with better outcomes, even for train users, accords with research that people trade off long commutes for compensations in other areas, such as better housing or higher salaries.^26^ This could drive some of the findings seen in these analyses.

The findings of this study of commuters in England and Wales are in line with existing evidence, in which switching to alternatives to car use is associated with health benefits.^4^, ^5^, ^6^ However, a systematic review found potential differences in the relation between studies in northern Europe and those from elsewhere, so the generalisability of our findings is uncertain.^4^ Differences in the built environment, public transport availability, and social norms might lead to these differences. Although more research is needed into the importance of contextual factors and the relative importance of the differing mechanisms at play, evidence is sufficient to support the policy aim of discouraging car use and encouraging alternative modes of transport.

There is considerable scope to increase the levels of active travel in England and Wales, with 61% of trips and 77% of distance travelled by car in 2018.^34^ Our study provides additional evidence for the health benefits associated with increased use of physically active travel modes, including public transport. Potential health benefits of public transport have received little attention previously, and our findings identified public transport use to be associated with a reduced rate of incident cancer, whereas a systematic review linked public transport use to reduced adiposity.^32^ These findings should inform policy decisions on future spending priorities, particularly those in transport and other non-health sectors. Increased walking, cycling, and public transport use would contribute to improved air quality and subsequent health benefits for all. Our findings also indicate that the associations between commute mode and health were consistent across occupation-based socioeconomic groups. If the relative health benefits of active travel apply to all, then the greater underlying risk experienced by people in lower socioeconomic groups would result in greater absolute benefits for these groups.^35^

By including up to 25 years of follow-up, our study adds to existing evidence for the beneficial health effects of physically active commute modes, including cycling and train use. These associations were similar across socioeconomic groups, strengthening calls for investment to encourage more physically active forms of travel. Further research on specific mechanisms behind these findings would be useful, but it should not detract from the large body of evidence that private car use will need to be reduced to meet future health and environmental goals.
