# Supplementary material for: Associations between commute mode and cardiovascular disease, cancer, and all-cause mortality, and cancer incidence, using linked Census data over 25 years in England and Wales: a cohort study
Source: Lancet Planet Health. 2020 May 19;4(5):e186–94. doi: 10.1016/S2542-5196(20)30079-6 (PMC7242898; doi:10.1016/S2542-5196(20)30079-6)
Supplement: Supplementary appendix [file mmc1.pdf]

THE LANCET
Planetary Health

Supplementary appendix

This appendix formed part of the original submission and has been peer reviewed. We post it as supplied by the authors.

Supplement to: Patterson R, Panter J, Vamos EP, Cummins S, Millett C, Laverty AA. Associations between commute mode and cardiovascular disease, cancer, and all-cause mortality, and cancer incidence, using linked Census data over 25 years in England and Wales: a cohort study. *Lancet Planet Health* 2020: **4:** e186–94.

List of Appendix Tables

Table 1: Commute mode options available in the census forms from 1991 to 2011 3
Table 2: International Classification of Diseases and Related Health Problems (ICD) codes used in Revision 9 and Revision 10 ... 3
Table 3: Comparison between those eligible for work who were included and excluded from the analyses 5
Table 4: Characteristics of participants in 1991 census by commute mode from ONS Longitudinal Study 6
Table 5: Characteristics of participants from 2001 census by commute mode from ONS Longitudinal Study 7
Table 6: Characteristics of participants in 2011 census by commute mode from ONS Longitudinal Study 8
Table 7: Baseline characteristics of individuals and commute mode with disaggregated public transport modes from ONS Longitudinal Study 1991-2016 9
Table 8: Cox regression analyses of commute mode and mortality and cancer outcomes using the ONS Longitudinal Study 1991-2016 10
Table 9: P-values for tests for interaction for each exposure and outcome combination 11
Table 10: NSSEC stratified Cox regression analyses of commute mode and mortality and cancer outcomes from the ONS Longitudinal Study 1991-2015/16 12
Table 11: Sex stratified Cox regression analyses of commute mode and mortality and cancer outcomes from the ONS Longitudinal Study 1991-2015/16 13
Table 12: straight line distance to work (km) among sample for 2001 and 2011 14
Table 13: Cox regression analyses of commute mode and mortality and cancer outcomes using the ONS Longitudinal Study 1991-2016 disaggregating long and short distance commuters. 15
Table 14: Baseline characteristics of sample and deaths 2001-2016 for participants in analyses of changes in mode between 1991 and 2001 – switching between private motorised vehicle and active or public transport and vice versa 16
Table 15: Cox regression analyses of change in commute mode between1991 and 2001 (active and public versus private motorised vehicle) and mortality and cancer outcomes from the ONS Longitudinal Study 2001-2015/16. 18
Table 16: Cox regression analyses of commute mode and mortality using the ONS Longitudinal Study 2001-2011 with and without adjustment for self-reported health 19
Table 17: Cox regression analyses of commute mode and mortality outcomes using the ONS Longitudinal Study 1991-2016, excluded the first 2 years of follow-up in each 10 year period 20
Table 18: Cross tabulation of commute modes for this present in consecutive censuses 21

Acknowledgement

This work contains statistical data from the Office for National Statistics (ONS) which is Crown Copyright. The use of the ONS statistical data in this work does not imply the endorsement of the ONS in relation to the interpretation or analysis of the statistical data. This work uses research datasets which may not exactly reproduce National Statistics aggregates.

The permission of the Office for National Statistics to use the Longitudinal Study is gratefully acknowledged, as is the help provided by staff of the Centre for Longitudinal Study Information & User Support (CeLSIUS). CeLSIUS is supported by the ESRC Census of Population Programme under project ES/R00823X/1. The authors alone are responsible for the interpretation of the data.

Table 1: Commute mode options available in the census forms from 1991 to 2011

	1991	2001	2011
Private motorised vehicle	Motor cycle, scooter, moped<br>Driving a car or van<br>Passenger in a car or van	Motor cycle, scooter or moped<br>Driving a car or van<br>Passenger in a car or van<br>Taxi	Motorcycle, scooter or moped<br>Driving a car or van<br>Passenger in a car or van<br>Taxi
Public Transport	Bus, minibus or coach (public or private)<br>Underground, tube, metro<br>British Rail train	Bus, minibus or coach<br>Underground, metro, light-rail, tram<br>Train	Bus, minibus or coach<br>Underground, metro, light rail, tram<br>Train
Cycle	Pedal cycle	Bicycle	Bicycle
Walk	On foot	On foot	On foot
Excluded		Work mainly at or from home	Work mainly at or from home
	Other please specify	Other	Other

Table 2: International Classification of Diseases and Related Health Problems (ICD) codes used in Revision 9 and Revision 10

	ICD-10	ICD-9
CVD (diseases of circulatory system)	I20-25 + I60-69	410-414 + 430-438
Neoplasms	C00-C97 + D37-84	140-209 +235-239

Figure 1: Flow diagram showing sample exclusions

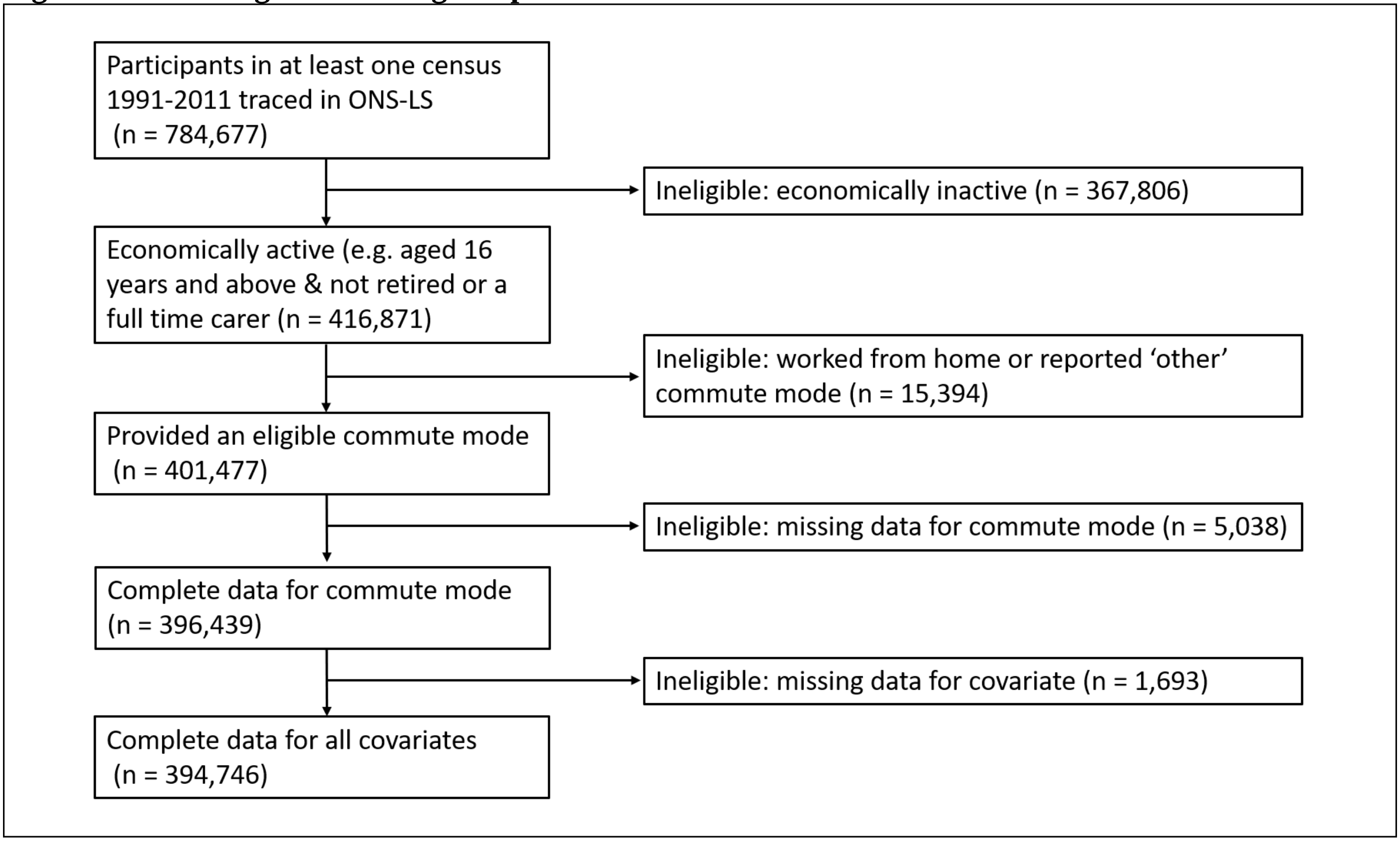

Table 3: Comparison between those eligible for work who were included and excluded from the analyses

	Sample		Excluded	
	N	%	N	%
All individuals	394746		6,731	
**Commute mode**				
Private motorised	258,665	65.5%	896	53.1%
Public Transport	74,377	18.8%	333	19.7%
Walk	49,024	12.4%	415	24.6%
Cycle	12,680	3.2%	44	2.6%
Total	394,746		1,688	
**Age**				
16-29	149,585	37.9%	883	26.7%
30-44	142,962	36.2%	705	21.3%
45-59	85,677	21.7%	1,212	36.6%
60+	16,522	4.2%	509	15.4%
Total	394,746		3,309	
**Sex**				
Male	209,510	53.1%	3,678	54.6%
Female	185,236	46.9%	3,053	45.4%
Total	394,746		6,731	
**Housing tenure**				
Homeowner	288,585	73.1%	1,708	67.3%
Non-homeowner	106,161	26.9%	831	32.7%
Total	394,746		2,539	
**Marital status**				
Unmarried	190,560	48.3%	1,495	45.3%
Married	204,186	51.7%	1,806	54.7%
Total	394,746		3,301	
**Ethnicity**				
White	353,842	89.6%	2,865	88.5%
Minority ethnicity	40,904	10.4%	374	11.5%
Total	394,746		3,239	
**University education**				
No degree	313,291	79.4%	3,007	92.5%
Has a degree	81,455	20.6%	243	7.5%
Total	394,746		3,250	
**Car access**				
No car access	53,953	13.7%	602	23.0%
Has access to a car	340,793	86.3%	2,010	77.0%
Total	394,746		2,612	
**Population density**				
0-2,000 persons/km2	172,332	43.7%	1,427	44.5%
2,000+ persons/km2	222,414	56.3%	1,777	55.5%
Total	394,746		3,204	
**NSSEC – social class**				
Higher	130,262	33.0%	871	32.3%
Intermediate	98,302	24.9%	786	29.1%
Routine/Manual	166,182	42.1%	1,043	38.6%
Total	394,746		2,700	
**Carstairs quintile**				
Least deprived	76,945	19.5%	425	18.6%
2	79,575	20.2%	372	16.2%
3	80,434	20.4%	406	17.7%
4	81,108	20.5%	487	21.3%
Most deprived	76,684	19.4%	601	26.2%
Total	394,746		2,291	
**Long term illness**				
No illness	376,939	95.5%	3,037	92.1%
Has an illness	17,807	4.5%	260	7.9%
Total	394,746		3,297	

NSSEC – National Statistics socio-economic classification
Source: Office for National Statistics (ONS) Longitudinal Study of England and Wales

Table 4: Characteristics of participants in 1991 census by commute mode from ONS Longitudinal Study

	Private motorised (%)	Public Transport (%)	Walk (%)	Cycle (%)	Total
**All**	68.4%	16.3%	12.0%	3.3%	209,209
**Age**					
16-29	64.2%	21.0%	11.5%	3.3%	61,480
30-44	72.0%	13.6%	11.2%	3.2%	79,233
45-59	68.8%	14.9%	13.0%	3.3%	57,506
60+	63.7%	16.8%	15.8%	3.8%	10,990
**Sex**					
Male	76.0%	12.5%	7.7%	3.8%	117,789
Female	58.6%	21.1%	17.6%	2.7%	91,420
**Housing tenure**					
Homeowner	72.6%	14.4%	10.0%	3.0%	169,125
Non-homeowner	50.7%	24.1%	20.7%	4.5%	40,084
**Marital status**					
Unmarried	61.4%	22.6%	12.6%	3.4%	74,156
Married	72.2%	12.8%	11.7%	3.2%	135,053
**Ethnicity**					
White	69.2%	15.3%	12.0%	3.4%	197,922
Minority ethnicity	53.8%	33.4%	11.8%	0.9%	11,287
**University education**					
No degree	67.9%	16.0%	12.7%	3.3%	188,526
Has a degree	72.7%	18.5%	5.8%	3.1%	20,683
**Car access**					
No car access	18.3%	44.7%	29.9%	7.1%	25,315
Has access to a car	75.3%	12.4%	9.6%	2.8%	183,894
**Population density**					
0-2,000 persons/km^2	75.8%	10.1%	10.9%	3.2%	100,130
2,000+ persons/km^2	61.5%	21.9%	13.1%	3.5%	109,079
**NSSEC – social class**					
Higher	78.3%	14.0%	5.6%	2.0%	62,588
Intermediate	71.6%	17.7%	8.7%	2.0%	55,611
Routine/Manual	59.6%	17.0%	18.4%	5.0%	91,010
**Carstairs quintile**					
Least deprived	79.4%	10.2%	7.3%	3.1%	45,127
2	74.3%	12.3%	10.2%	3.2%	44,602
3	69.0%	14.7%	12.7%	3.7%	43,240
4	62.8%	18.9%	14.7%	3.6%	41,601
Most deprived	52.3%	28.2%	16.5%	3.0%	34,639
**Long term illness**					
No illness	68.5%	16.2%	12.0%	3.3%	202,200
Has an illness	65.3%	18.6%	13.1%	2.9%	7,009
**Events (1991-2001)**					
All-cause mortality	66.1%	17.1%	13.7%	3.1%	6,169
CVD mortality	68.8%	15.5%	12.5%	3.2%	1,707
Cancer incidence	68.5%	14.7%	13.8%	3.0%	7,083
Cancer mortality	61.9%	20.0%	14.8%	3.3%	2,733

NSSEC – National Statistics socio-economic classification
Source: Office for National Statistics (ONS) Longitudinal Study of England and Wales

Table 5: Characteristics of participants from 2001 census by commute mode from ONS Longitudinal Study

	Private motorised (%)	Public Transport (%)	Walk (%)	Cycle (%)	Total
**All**	72.1%	14.7%	10.2%	3.0%	209,371
**Age**					
16-29	63.9%	21.7%	11.2%	3.2%	47,295
30-44	74.3%	13.4%	9.1%	3.2%	84,651
45-59	75.0%	11.7%	10.6%	2.6%	66,528
60+	72.4%	12.4%	12.4%	2.8%	10,897
**Sex**					
Male	76.9%	12.3%	6.8%	4.0%	111,755
Female	66.6%	17.4%	14.2%	1.8%	97,616
**Housing tenure**					
Homeowner	76.0%	12.8%	8.5%	2.7%	170,369
Non-homeowner	55.1%	23.0%	17.7%	4.2%	39,002
**Marital status**					
Unmarried	65.8%	19.7%	11.2%	3.3%	90,958
Married	76.9%	10.9%	9.4%	2.8%	118,413
**Ethnicity**					
White	73.1%	13.6%	10.2%	3.1%	194,860
Minority ethnicity	58.5%	29.9%	10.4%	1.2%	14,511
**University education**					
No degree	72.1%	13.5%	11.3%	3.0%	159,300
Has a degree	72.0%	18.6%	6.6%	2.8%	50,071
**Car access**					
No car access	19.9%	44.6%	28.1%	7.4%	19,458
Has access to a car	77.4%	11.6%	8.4%	2.5%	189,913
**Population density**					
0-2,000 persons/km^2	79.8%	8.5%	9.1%	2.6%	100,134
2,000+ persons/km^2	65.0%	20.4%	11.2%	3.3%	109,237
**NSSEC – social class**					
Higher	76.0%	16.0%	5.8%	2.2%	82,367
Intermediate	74.9%	15.3%	8.2%	1.6%	44,524
Routine/Manual	66.7%	13.1%	15.7%	4.5%	82,480
**Carstairs quintile**					
Least deprived	82.7%	8.9%	6.1%	2.3%	45,498
2	77.3%	11.1%	8.6%	3.0%	45,447
3	72.2%	13.2%	11.3%	3.3%	43,900
4	68.1%	16.1%	12.5%	3.3%	40,702
Most deprived	55.5%	27.7%	13.8%	3.1%	33,824
**Long term illness**					
No illness	72.2%	14.7%	10.1%	3.0%	195,225
Has an illness	71.1%	14.5%	11.7%	2.7%	14,146
**Events (2001-2011)**					
All-cause mortality	71.5%	13.3%	12.4%	2.8%	4,963
CVD mortality	74.5%	12.0%	11.0%	2.5%	1,000
Cancer incidence	75.2%	11.6%	10.4%	2.7%	8,806
Cancer mortality	68.1%	15.7%	13.4%	2.9%	2,381

NSSEC – National Statistics socio-economic classification
Source: Office for National Statistics (ONS) Longitudinal Study of England and Wales

Table 6: Characteristics of participants in 2011 census by commute mode from ONS Longitudinal Study

	Private motorised (%)	Public Transport (%)	Walk (%)	Cycle (%)	Total
**All**	69.9%	16.6%	10.6%	2.9%	247,074
**Age**					
16-29	60.4%	23.0%	13.6%	3.0%	51,542
30-44	69.8%	17.6%	9.4%	3.3%	88,770
45-59	74.7%	12.7%	10.0%	2.6%	83,873
60+	74.6%	12.5%	11.0%	2.0%	22,889
**Sex**					
Male	73.6%	14.8%	7.7%	3.9%	129,675
Female	65.9%	18.6%	13.8%	1.7%	117,399
**Housing tenure**					
Homeowner	75.6%	13.5%	8.4%	2.6%	181,000
Non-homeowner	54.4%	25.1%	16.8%	3.7%	66,074
**Marital status**					
Unmarried	64.3%	20.3%	12.2%	3.1%	118,312
Married	75.1%	13.2%	9.1%	2.6%	128,762
**Ethnicity**					
White	72.2%	14.2%	10.5%	3.1%	215,897
Minority ethnicity	54.1%	33.1%	11.3%	1.5%	31,177
**University education**					
No degree	71.2%	14.1%	12.1%	2.6%	161,350
Has a degree	67.5%	21.3%	7.9%	3.3%	85,724
**Car access**					
No car access	17.2%	49.5%	26.6%	6.7%	26,693
Has access to a car	76.3%	12.6%	8.7%	2.4%	220,381
**Population density**					
0-2,000 persons/km^2	80.3%	8.6%	9.0%	2.1%	106,563
2,000+ persons/km^2	62.1%	22.6%	11.9%	3.4%	140,511
**NSSEC – social class**					
Higher	70.6%	19.2%	7.2%	2.9%	97,389
Intermediate	73.7%	14.8%	9.7%	1.8%	63,203
Routine/Manual	66.4%	14.9%	15.1%	3.6%	86,482
**Carstairs quintile**					
Least deprived	80.2%	10.4%	7.1%	2.3%	50,606
2	75.6%	12.8%	8.9%	2.8%	52,309
3	71.5%	14.1%	11.4%	3.0%	51,105
4	66.3%	18.1%	12.5%	3.1%	49,038
Most deprived	53.5%	29.5%	13.7%	3.2%	44,016
**Long term illness**					
No illness	69.9%	16.7%	10.5%	2.9%	229,114
Has an illness	70.8%	15.8%	11.4%	2.0%	17,960
**Events (2011-2015/16)**					
All-cause mortality	71.5%	14.8%	11.1%	2.6%	2,851
CVD mortality	70.9%	16.5%	9.4%	3.2%	467
Cancer incidence	75.4%	12.7%	9.7%	2.2%	5,094
Cancer mortality	70.3%	16.3%	10.9%	2.5%	1,399

NSSEC – National Statistics socio-economic classification
Source: Office for National Statistics (ONS) Longitudinal Study of England and Wales

Table 7: Baseline characteristics of individuals and commute mode with disaggregated public transport modes from ONS Longitudinal Study 1991-2016

	Private motorised vehicle (%)	Bus (%)	Rail (%)	Walk (%)	Cycle (%)	Total
**All**	65.5%	10.3%	8.5%	12.4%	3.2%	394,746
**Age**						
16-29	61.9%	12.2%	10.4%	12.3%	3.2%	149,593
30-44	68.0%	8.3%	8.8%	11.6%	3.3%	142,958
45-59	67.8%	10.2%	5.4%	13.4%	3.0%	85,674
60+	64.3%	11.3%	5.5%	15.7%	3.2%	16,521
**Sex**						
Men	71.6%	6.9%	8.7%	8.7%	4.0%	209,510
Women	58.7%	14.1%	8.3%	16.6%	2.3%	185,236
**Housing tenure**						
Homeowner	71.3%	8.2%	7.6%	10.0%	2.9%	288,584
Non-homeowner	49.8%	16.0%	11.1%	18.9%	4.2%	106,162
**Marital status**						
Unmarried	60.0%	12.8%	10.8%	13.0%	3.4%	190,566
Married	70.7%	8.0%	6.4%	11.9%	3.0%	204,180
**Ethnicity**						
White	67.2%	9.4%	7.5%	12.4%	3.4%	353,842
Minority ethnicity	50.9%	17.7%	17.5%	12.5%	1.4%	40,904
**University education**						
No degree	66.3%	11.2%	6.0%	13.4%	3.2%	313,292
Has a degree	62.5%	6.9%	18.5%	8.7%	3.4%	81,454
**Car access**						
No car access	17.9%	29.9%	17.6%	27.9%	6.7%	53,952
Has access to a car	73.1%	7.2%	7.1%	10.0%	2.7%	340,794
**Population density**						
0-2,000 persons/km^2	75.3%	6.5%	4.1%	11.2%	2.9%	172,330
2,000+ persons/km^2	57.9%	13.2%	12.0%	13.4%	3.5%	222,416
**NSSEC – social class**						
Higher	70.4%	5.9%	13.8%	7.3%	2.6%	130,264
Intermediate	69.4%	10.0%	9.1%	9.6%	1.9%	98,298
Routine/Manual	59.4%	13.9%	4.1%	18.1%	4.5%	166,184
**Carstairs quintile**						
Least deprived	78.1%	4.3%	6.7%	8.0%	2.8%	76,946
2	72.4%	6.6%	7.3%	10.6%	3.1%	79,574
3	66.7%	9.0%	7.6%	13.2%	3.5%	80,434
4	61.0%	12.7%	8.2%	14.6%	3.4%	81,109
Most deprived	49.3%	19.0%	13.0%	15.6%	3.2%	76,683
**Long term illness**						
No illness	65.6%	10.1%	8.6%	12.3%	3.2%	376,939
Has an illness	63.1%	13.6%	6.4%	14.3%	2.7%	17,807
**Entered study**						
1991	68.4%	9.9%	6.4%	12.0%	3.3%	209,217
2001	65.2%	10.3%	9.0%	12.4%	3.1%	88,633
2011	59.6%	11.2%	12.8%	13.3%	3.1%	96,896
**Events**						
All-cause mortality	67.8%	10.5%	5.4%	13.1%	3.1%	13,983
CVD mortality	69.9%	9.8%	5.2%	12.1%	2.9%	3,172
Cancer incidence	73.0%	8.0%	4.9%	11.4%	2.7%	20,980
Cancer mortality	64.4%	12.0%	6.2%	14.0%	3.5%	6,509

NSSEC – National Statistics socio-economic classification
Source: Office for National Statistics (ONS) Longitudinal Study of England and Wales

Table 8: Cox regression analyses of commute mode and mortality and cancer outcomes using the ONS Longitudinal Study 1991-2016

	**All-cause mortality**		**CVD mortality**		**Cancer incidence**		**Cancer mortality**	
**Commute mode**	**Unadjusted HR (95%CI)**	**Adjusted HR (95%CI)**	**Unadjusted HR (95%CI)**	**Adjusted HR (95%CI)**	**Unadjusted HR (95%CI)**	**Adjusted HR (95%CI)**	**Unadjusted HR (95%CI)**	**Adjusted HR (95%CI)**
Private motorised	1.00	1.00	1.00	1.00	1.00	1.00	1.00	1.00
Public transport	0.98 (0.94, 1.03)	0.96 (0.91, 1.02)	0.91 (0.82, 1.00)	0.90 (0.80, 1.01)	0.79 (0.76, 0.82)	0.93 (0.89, 0.97)	0.89 (0.83, 0.96)	0.95 (0.88, 1.03)
Walk	1.17 (1.11, 1.23)	0.98 (0.92, 1.03)	1.02 (0.91, 1.14)	0.91 (0.81, 1.03)	1.00 (0.96, 1.05)	0.93 (0.89, 0.97)	1.16 (1.08, 1.25)	0.98 (0.91, 1.06)
Cycle	0.94 (0.85, 1.03)	0.80 (0.73, 0.89)	0.93 (0.75, 1.14)	0.76 (0.61, 0.93)	0.84 (0.78, 0.92)	0.89 (0.82, 0.97)	0.88 (0.76, 1.03)	0.84 (0.73, 0.98)
	Failures=13983	N=394,746	Failures=3172	N=394,746	Failures=20,980	N=393410	Failures=6,509	N=394,746

Disaggregated public transport modes

	**All-cause mortality**		**CVD mortality**		**Cancer incidence**		**Cancer mortality**	
**Commute mode**	**Unadjusted HR (95%CI)**	**Adjusted HR (95%CI)**	**Unadjusted HR (95%CI)**	**Adjusted HR (95%CI)**	**Unadjusted HR (95%CI)**	**Adjusted HR (95%CI)**	**Unadjusted HR (95%CI)**	**Adjusted HR (95%CI)**
Private motorised	1.00	1.00	1.00	1.00	1.00	1.00	1.00	1.00
Bus	1.19 (1.13, 1.26)	1.01 (0.94, 1.07)	1.11 (0.99, 1.25)	0.97 (0.84, 1.11)	0.92 (0.88, 0.97)	0.96 (0.91, 1.01)	1.10 (1.01, 1.19)	0.99 (0.90, 1.08)
Rail	0.73 (0.68, 0.79)	0.90 (0.83, 0.97)	0.65 (0.55, 0.77)	0.79 (0.67, 0.94)	0.64 (0.60, 0.68)	0.88 (0.83, 0.94)	0.66 (0.58, 0.74)	0.90 (0.80, 1.01)
Walk	1.17 (1.11, 1.23)	0.98 (0.93, 1.04)	1.02 (0.91, 1.14)	0.92 (0.82, 1.04)	1.01 (0.96, 1.05)	0.93 (0.89, 0.97)	1.16 (1.08, 1.25)	0.99 (0.91, 1.07)
Cycle	0.94 (0.85, 1.04)	0.81 (0.73, 0.89)	0.93 (0.75, 1.14)	0.76 (0.62, 0.94)	0.84 (0.78, 0.92)	0.89 (0.82, 0.97)	0.88 (0.76, 1.03)	0.85 (0.73, 0.99)
	Failures=13983	N=394,746	Failures=3172	N=394,746	Failures=20,980	N=393410	Failures=6,509	N=394,746

*Adjusted for: age at baseline; age at baseline squared; sex; ethnicity (minority ethnic group, white); housing tenure (homeowner, non-homeowner); marital status (married, non-married); University education (no degree, has a degree); access to a car (yes, no); National Statistics Social-economic Classification (higher, intermediate, routine/manual); year entered study and presence of a long term illness at baseline (yes, no), neighbourhood measures included were population density (<2000 persons per km^2, 2000+ persons per km^2) and individual level Carstairs quintile (a composite of male unemployment, lack of car ownership, overcrowding and social class of household head)

Source: Office for National Statistics (ONS) Longitudinal Study of England and Wales

Table 9: P-values for tests for interaction for each exposure and outcome combination

P-values	Gender (male/female)	population density (<2000/ 2000+ persons/km^2)	NSSEC social classification (routine/ intermediate/ managerial)	part-time working (part-time/ full-time)*
**All-cause mortality**				
Mode	0.022	0.2757	0.025	0.478
Disaggregated mode	0.002	0.355	0.001	0.435
**CVD mortality**				
Mode	0.159	0.413	0.704	0.879
Disaggregated mode	0.221	0.549	0.086	0.595
**Cancer incidence**				
Mode	0.044	0.585	0.110	0.219
Disaggregated mode	0.023	0.667	0.287	0.203
**Cancer mortality**				
Mode	0.086	0.639	0.249	0.910
Disaggregated mode	0.057	0.781	0.364	0.971

Source: Office for National Statistics (ONS) Longitudinal Study of England and Wales
Mode categorised as: Private motorised vehicle, public transport, walk, cycle
Disaggregated mode categorised as: Private motorised vehicle, bus rail, walk, cycle
*carried out on data from 2001 and 2011 only.

Table 10: NSSEC stratified Cox regression analyses of commute mode and mortality and cancer outcomes from the ONS Longitudinal Study 1991-2015/16

Managerial occupations

	**All-cause mortality**		**CVD mortality**		**Cancer incidence**		**Cancer mortality**	
**Commute mode**	**Unadjusted HR (95%CI)**	**Adjusted HR (95%CI)**	**Unadjusted HR (95%CI)**	**Adjusted HR (95%CI)**	**Unadjusted HR (95%CI)**	**Adjusted HR (95%CI)**	**Unadjusted HR (95%CI)**	**Adjusted HR (95%CI)**
Private motorised	1.00	1.00	1.00	1.00	1.00	1.00	1.00	1.00
Bus	1.14 (0.99, 1.31)	0.95 (0.82, 1.11)	1.20 (0.89, 1.61)	1.08 (0.77, 1.50)	0.81 (0.72, 0.91)	0.94 (0.83, 1.07)	0.89 (0.71, 1.11)	0.81 (0.64, 1.03)
Rail	0.65 (0.58, 0.74)	0.76 (0.67, 0.87)	0.49 (0.36, 0.66)	0.54 (0.39, 0.74)	0.64 (0.59, 0.70)	0.92 (0.84, 1.00)	0.64 (0.54, 0.76)	0.84 (0.71, 1.01)
Walk	1.09 (0.96, 1.24)	0.98 (0.86, 1.12)	0.92 (0.68, 1.23)	0.86 (0.63, 1.18)	0.82 (0.74, 0.91)	0.89 (0.80, 0.99)	1.05 (0.87, 1.26)	0.99 (0.82, 1.20)
Cycle	0.64 (0.49, 0.83)	0.71 (0.55, 0.92)	0.73 (0.43, 1.24)	0.80 (0.47, 1.37)	0.72 (0.61, 0.85)	0.93 (0.78, 1.11)	0.69 (0.48, 0.97)	0.86 (0.60, 1.22)
	Failures = 3,833	N = 168231	Failures = 794	N = 168231	Failures = 7,148	N = 167056	Failures = 1,897	N = 168231

Intermediate occupations

	**All-cause mortality**		**CVD mortality**		**Cancer incidence**		**Cancer mortality**	
**Commute mode**	**Unadjusted HR (95%CI)**	**Adjusted HR (95%CI)**	**Unadjusted HR (95%CI)**	**Adjusted HR (95%CI)**	**Unadjusted HR (95%CI)**	**Adjusted HR (95%CI)**	**Unadjusted HR (95%CI)**	**Adjusted HR (95%CI)**
Private motorised	1.00	1.00	1.00	1.00	1.00	1.00	1.00	1.00
Bus	0.98 (0.86, 1.10)	1.06 (0.92, 1.22)	0.84 (0.64, 1.12)	1.16 (0.84, 1.60)	0.81 (0.72, 0.90)	0.92 (0.82, 1.04)	0.94 (0.79, 1.13)	1.03 (0.84, 1.26)
Rail	0.72 (0.62, 0.84)	0.90 (0.77, 1.05)	0.65 (0.46, 0.91)	0.88 (0.62, 1.25)	0.61 (0.53, 0.69)	0.85 (0.74, 0.97)	0.59 (0.47, 0.74)	0.81 (0.64, 1.04)
Walk	1.17 (1.05, 1.31)	1.13 (1.00, 1.27)	1.04 (0.80, 1.34)	1.12 (0.85, 1.46)	1.07 (0.98, 1.18)	1.03 (0.94, 1.14)	1.21 (1.03, 1.42)	1.14 (0.97, 1.35)
Cycle	0.81 (0.61, 1.07)	0.84 (0.63, 1.11)	0.77 (0.41, 1.43)	0.86 (0.46, 1.62)	0.71 (0.56, 0.90)	0.80 (0.63, 1.01)	0.78 (0.52, 1.18)	0.86 (0.57, 1.30)
	Failures = 3,277	N = 125614	Failures = 690	N = 125614	Failures = 5,163	N = 124645	Failures = 1,609	N = 125614

Routine/manual occupations

	**All-cause mortality**		**CVD mortality**		**Cancer incidence**		**Cancer mortality**	
**Commute mode**	**Unadjusted HR (95%CI)**	**Adjusted HR (95%CI)**	**Unadjusted HR (95%CI)**	**Adjusted HR (95%CI)**	**Unadjusted HR (95%CI)**	**Adjusted HR (95%CI)**	**Unadjusted HR (95%CI)**	**Adjusted HR (95%CI)**
Private motorised	1.00	1.00	1.00	1.00	1.00	1.00	1.00	1.00
Bus	1.16 (1.08, 1.24)	1.03 (0.95, 1.12)	1.01 (0.87, 1.17)	0.90 (0.76, 1.06)	1.01 (0.94, 1.08)	0.99 (0.92, 1.07)	1.14 (1.02, 1.27)	1.06 (0.93, 1.19)
Rail	1.13 (0.99, 1.28)	1.10 (0.96, 1.25)	1.18 (0.92, 1.51)	1.03 (0.79, 1.33)	0.70 (0.61, 0.81)	0.87 (0.75, 1.00)	0.94 (0.76, 1.17)	1.08 (0.87, 1.34)
Walk	1.04 (0.98, 1.11)	0.96 (0.90, 1.04)	0.86 (0.75, 0.99)	0.86 (0.74, 1.01)	1.03 (0.97, 1.09)	0.93 (0.88, 0.99)	1.09 (0.99, 1.20)	0.97 (0.87, 1.08)
Cycle	0.96 (0.86, 1.08)	0.83 (0.73, 0.93)	0.86 (0.68, 1.10)	0.73 (0.57, 0.94)	0.93 (0.84, 1.03)	0.90 (0.81, 1.01)	0.93 (0.77, 1.11)	0.84 (0.70, 1.01)
	Failures = 6,873	N = 185273	Failures = 1,688	N = 185273	Failures = 8,669	N = 184304	Failures = 3,003	N = 185273

*Adjusted for: age at baseline; age at baseline squared; sex; ethnicity (minority ethnic group, white); housing tenure (homeowner, non-homeowner); marital status (married, non-married); University education (no degree, has a degree); access to a car (yes, no); year entered study and presence of a long term illness at baseline (yes, no), neighbourhood measures included were population density (<2000 persons per km2, 2000+ persons per km^2) and individual level Carstairs quintile (a composite of male unemployment, lack of car ownership, overcrowding and social class of household head)

Source: Office for National Statistics (ONS) Longitudinal Study of England and Wales

Table 11: Sex stratified Cox regression analyses of commute mode and mortality and cancer outcomes from the ONS Longitudinal Study 1991-2015/16

Males

	**All-cause mortality**		**CVD mortality**		**Cancer incidence**		**Cancer mortality**	
	**Unadjusted**	**Adjusted**	**Unadjusted**	**Adjusted**	**Unadjusted**	**Adjusted**	**Unadjusted**	**Adjusted**
**Commute mode**	**HR (95%CI)**	**HR (95%CI)**	**HR (95%CI)**	**HR (95%CI)**	**HR (95%CI)**	**HR (95%CI)**	**HR (95%CI)**	**HR (95%CI)**
Private motorised	1.00	1.00	1.00	1.00	1.00	1.00	1.00	1.00
Bus	1.41 (1.31, 1.53)	1.08 (0.99, 1.18)	1.41 (1.22, 1.64)	1.02 (0.87, 1.21)	0.86 (0.78, 0.93)	1.02 (0.92, 1.12)	1.16 (1.02, 1.32)	1.09 (0.94, 1.26)
Rail	0.75 (0.69, 0.82)	0.90 (0.82, 0.99)	0.64 (0.53, 0.77)	0.78 (0.64, 0.94)	0.67 (0.62, 0.73)	0.91 (0.84, 0.99)	0.68 (0.59, 0.79)	0.94 (0.81, 1.09)
Walk	1.24 (1.15, 1.33)	1.01 (0.94, 1.09)	1.09 (0.95, 1.26)	0.88 (0.75, 1.02)	0.90 (0.84, 0.97)	0.99 (0.92, 1.07)	1.14 (1.02, 1.28)	1.07 (0.95, 1.20)
Cycle	0.82 (0.73, 0.92)	0.80 (0.71, 0.90)	0.79 (0.63, 0.99)	0.78 (0.62, 0.99)	0.77 (0.69, 0.86)	0.93 (0.84, 1.04)	0.75 (0.63, 0.91)	0.84 (0.70, 1.02)
	Failures = 9363	N = 209510	Failures = 2517	N = 209510	Failures = 11174	N = 209024	Failures = 3869	N = 209510

Female

	**All-cause mortality**		**CVD mortality**		**Cancer incidence**		**Cancer mortality**	
	**Unadjusted**	**Adjusted**	**Unadjusted**	**Adjusted**	**Unadjusted**	**Adjusted**	**Unadjusted**	**Adjusted**
**Commute mode**	**HR (95%CI)**	**HR (95%CI)**	**HR (95%CI)**	**HR (95%CI)**	**HR (95%CI)**	**HR (95%CI)**	**HR (95%CI)**	**HR (95%CI)**
Private motorised	1.00	1.00	1.00	1.00	1.00	1.00	1.00	1.00
Bus	1.38 (1.27, 1.50)	0.95 (0.87, 1.04)	1.80 (1.46, 2.23)	0.88 (0.69, 1.12)	0.96 (0.90, 1.02)	0.96 (0.90, 1.03)	1.20 (1.07, 1.34)	0.96 (0.84, 1.08)
Rail	0.72 (0.62, 0.83)	0.89 (0.77, 1.03)	0.82 (0.56, 1.19)	0.93 (0.63, 1.38)	0.60 (0.55, 0.66)	0.84 (0.76, 0.93)	0.62 (0.51, 0.75)	0.84 (0.69, 1.03)
Walk	1.46 (1.35, 1.57)	0.97 (0.89, 1.05)	1.99 (1.65, 2.41)	0.96 (0.78, 1.19)	1.06 (1.01, 1.12)	0.94 (0.88, 1.00)	1.32 (1.20, 1.46)	0.98 (0.88, 1.09)
Cycle	1.20 (0.98, 1.45)	0.85 (0.70, 1.03)	1.39 (0.84, 2.30)	0.78 (0.47, 1.29)	1.01 (0.88, 1.16)	0.88 (0.77, 1.01)	1.19 (0.93, 1.54)	0.91 (0.70, 1.17)
	Failures = 4630	N = 185236	Failures = 657	N = 185236	Failures = 9806	N = 184386	Failures = 2644	N = 185236

*Adjusted for: age at baseline; age at baseline squared; ethnicity (minority ethnic group, white); housing tenure (homeowner, non-homeowner); marital status (married, non-married); University education (no degree, has a degree); access to a car (yes, no); year entered study and presence of a long term illness at baseline (yes, no), neighbourhood measures included were population density (<2000 persons per km2, 2000+ persons per km^2), National Statistics Social-economic Classification (higher, intermediate, routine/manual) and individual level Carstairs quintile (a composite of male unemployment, lack of car ownership, overcrowding and social class of household head)
Source: Office for National Statistics (ONS) Longitudinal Study of England and Wales

Table 12: straight line distance to work (km) among sample for 2001 and 2011

		N	Median	25th percentile	75th percentile	Mean	SD
Private motorised vehicle		307442	6.5	2.7	14.3	13.9	29.5
Bus		36918	4.4	2.6	7.4	8.8	25.1
Rail		37231	13.1	7.4	24.5	23.8	36.8
Walk		52514	0.8	0.3	1.5	4.8	26.5
Cycle		13834	2.5	1.3	4.8	5.9	21.5
**Long and short distance commuters**							
Private motorised vehicle	Short	155491	2.8	1.4	4.4	2.9	1.9
Private motorised vehicle	Long	151951	14.5	9.7	24.3	25.2	38.8
Bus	Short	18846	2.6	1.8	3.5	2.6	1.1
Bus	Long	18072	7.5	5.7	11.5	15.3	34.7
Rail	Short	19233	7.6	4.7	10.4	7.4	3.7
Rail	Long	17998	25.4	17.6	46.7	41.5	46.8
Walk	Short	27364	0.3	0.1	0.6	0.4	0.2
Walk	Long	25150	1.6	1.1	2.8	9.7	37.7
Cycle	Short	7097	1.3	0.8	1.9	1.3	0.7
Cycle	Long	6737	4.9	3.5	7.8	10.8	30.0

Source: Office for National Statistics (ONS) Longitudinal Study of England and Wales

Table 13: Cox regression analyses of commute mode and mortality and cancer outcomes using the ONS Longitudinal Study 1991-2016 disaggregating long and short distance commuters.

		**All-cause mortality**		**CVD mortality**		**Cancer incidence**		**Cancer mortality**	
		**Unadjusted**	**Adjusted**	**Unadjusted**	**Adjusted**	**Unadjusted**	**Adjusted**	**Unadjusted**	**Adjusted**
**Commute mode**		**HR (95%CI)**	**HR (95%CI)**	**HR (95%CI)**	**HR (95%CI)**	**HR (95%CI)**	**HR (95%CI)**	**HR (95%CI)**	**HR (95%CI)**
Private motorised	Short	1.00	1.00	1.00	1.00	1.00	1.00	1.00	1.00
Private motorised	Long	0.87 (0.82, 0.92)	0.97 (0.92, 1.03)	0.94 (0.83, 1.07)	1.03 (0.90, 1.17)	0.90 (0.87, 0.94)	0.99 (0.95, 1.03)	0.83 (0.77, 0.90)	0.96 (0.88, 1.04)
Bus	Short	1.21 (1.09, 1.35)	1.02 (0.91, 1.15)	1.27 (0.99, 1.63)	1.19 (0.91, 1.55)	0.88 (0.80, 0.96)	0.95 (0.86, 1.04)	1.06 (0.90, 1.24)	0.96 (0.80, 1.14)
Bus	Long	0.99 (0.88, 1.12)	0.97 (0.85, 1.10)	0.97 (0.73, 1.29)	0.98 (0.73, 1.32)	0.77 (0.70, 0.85)	0.97 (0.88, 1.08)	0.86 (0.72, 1.03)	0.94 (0.78, 1.14)
Rail	Short	0.68 (0.59, 0.79)	0.94 (0.81, 1.09)	0.65 (0.46, 0.91)	0.90 (0.63, 1.29)	0.57 (0.51, 0.63)	0.92 (0.82, 1.04)	0.61 (0.49, 0.75)	0.97 (0.78, 1.21)
Rail	Long	0.54 (0.46, 0.63)	0.80 (0.68, 0.94)	0.43 (0.28, 0.65)	0.65 (0.42, 0.98)	0.61 (0.54, 0.68)	0.87 (0.78, 0.97)	0.52 (0.42, 0.66)	0.84 (0.66, 1.05)
Walk	Short	1.20 (1.10, 1.32)	1.01 (0.91, 1.11)	1.12 (0.90, 1.41)	1.04 (0.83, 1.31)	1.01 (0.94, 1.09)	0.92 (0.86, 1.00)	1.23 (1.08, 1.41)	1.04 (0.90, 1.19)
Walk	Long	0.98 (0.88, 1.09)	0.95 (0.85, 1.06)	0.86 (0.66, 1.12)	0.91 (0.69, 1.19)	0.75 (0.69, 0.82)	0.86 (0.78, 0.94)	0.92 (0.79, 1.07)	0.95 (0.81, 1.11)
Cycle	Short	0.93 (0.77, 1.12)	0.83 (0.69, 1.01)	0.82 (0.52, 1.32)	0.72 (0.45, 1.16)	0.82 (0.70, 0.95)	0.89 (0.77, 1.03)	0.89 (0.68, 1.18)	0.89 (0.67, 1.17)
Cycle	Long	0.78 (0.64, 0.97)	0.80 (0.65, 0.99)	0.85 (0.53, 1.36)	0.82 (0.51, 1.32)	0.75 (0.65, 0.88)	0.98 (0.83, 1.14)	0.77 (0.57, 1.04)	0.93 (0.69, 1.26)
		Failures=7146	N=306578	Failures=1327	N=306578	Failures=12896	N=303151	Failures=3464	N=306578

*Adjusted for: age at baseline; age at baseline squared; sex; ethnicity (minority ethnic group, white); housing tenure (homeowner, non-homeowner); marital status (married, non-married); University education (no degree, has a degree); access to a car (yes, no); National Statistics Social-economic Classification (higher, intermediate, routine/manual); year entered study and presence of a long term illness at baseline (yes, no), neighbourhood measures included were population density (<2000 persons per km^2, 2000+ persons per km^2) and individual level Carstairs quintile (a composite of male unemployment, lack of car ownership, overcrowding and social class of household head)

Source: Office for National Statistics (ONS) Longitudinal Study of England and Wales

Table 14: Baseline characteristics of sample and deaths 2001-2016 for participants in analyses of changes in mode between 1991 and 2001 – switching between private motorised vehicle and active or public transport and vice versa

	Continuing car user	Switched from car to active or public transport	Continuing active or public transport user	Switched from active or public transport to car	Total
	N (%)	N (%)	N (%)	N (%)	
All	78.6%	5.3%	7.2%	8.9%	96,939
**Age**					
0-29	77.2%	6.3%	4.9%	11.6%	31,616
30-44	79.6%	4.8%	7.6%	8.0%	46,708
45-59	78.3%	4.8%	10.4%	6.6%	18,173
60+	74.4%	7.5%	12.2%	5.9%	442
**Sex**					
Male	82.9%	4.9%	4.8%	7.4%	57,964
Female	72.1%	5.9%	10.8%	11.2%	38,975
**Ethnicity**					
White	78.5%	5.3%	7.3%	8.9%	93,524
Minority ethnicity	80.8%	4.2%	5.7%	9.3%	3,415
**University education**					
No degree	76.7%	5.6%	8.2%	9.4%	75,611
Has a degree	85.1%	4.1%	3.7%	7.1%	21,328
**Housing tenure**					
Owns in both	81.7%	4.7%	6.1%	7.5%	77,923
Becomes a renter	70.8%	11.2%	6.8%	11.2%	4,084
Rents in both	57.2%	8.3%	20.4%	14.1%	6,109
Becomes an owner	69.4%	5.8%	8.0%	16.8%	8,823
**Marital status**					
Unmarried in both	74.7%	5.9%	8.4%	11.0%	19,809
Becomes married	79.4%	6.0%	3.9%	10.7%	13,365
Married in both	79.9%	4.7%	7.7%	7.7%	55,459
Becomes unmarried	77.5%	6.4%	7.1%	8.9%	8,306
**Car access**					
No access at either	13.6%	9.1%	64.3%	13.0%	1,903
Gains access	35.9%	4.6%	19.5%	40.0%	3,891
Access in both	82.6%	4.8%	5.0%	7.5%	89,567
Loses access	32.0%	26.7%	34.0%	7.4%	1,578
**Population density**					
Low density in both	81.3%	4.7%	6.5%	7.6%	41,870
Moves from low to high density	77.7%	7.3%	5.4%	9.6%	9,123
High density in both density	75.2%	5.6%	9.5%	9.7%	34,582
Moves from high to low	79.6%	4.7%	4.6%	11.1%	11,364
**Long term illness**					
Never ill	78.7%	5.3%	7.2%	8.9%	88,751
Becomes ill	76.9%	5.6%	8.2%	9.3%	6,304
Ill in both	80.7%	4.4%	7.7%	7.2%	867
Becomes well	76.1%	6.1%	7.9%	9.9%	1,017
**Baseline NSSEC social class**					
Higher	87.9%	3.7%	3.1%	5.2%	31,005
Intermediate	83.0%	5.1%	4.6%	7.3%	25,509
Routine/Manual	68.6%	6.6%	12.1%	12.8%	40,425
**Change in NSSEC social class**					
Unchanged NSSEC	78.0%	5.1%	8.4%	8.5%	65,677
Increased NSSEC	79.0%	4.7%	4.8%	11.5%	20,014
Decreased NSSEC	80.8%	7.4%	4.8%	7.0%	11,248
**Baseline Carstairs index**					
Least deprived	84.9%	4.6%	4.3%	6.3%	22,553
Next least deprived	80.8%	5.2%	6.1%	7.9%	22,123
Mid-point	77.0%	5.6%	8.2%	9.3%	20,991
Moderate deprivation	74.8%	5.6%	8.8%	10.7%	18,680
Most deprived	71.5%	5.6%	10.7%	12.2%	12,592
**Change in Carstairs index**					

Unchanged deprivation quintile	78.6%	5.0%	8.2%	8.2%	47,934
Moved to a more deprived quintile	79.3%	4.9%	5.8%	10.1%	27,053
Moved to a less deprived quintile	77.5%	6.4%	6.9%	9.1%	21,952
**Residential address**					
Did not move	77.6%	4.6%	10.2%	7.6%	46,997
Moved residence	79.4%	5.9%	4.4%	10.2%	49,915
**Events**					
All-cause mortality	78.4%	5.0%	9.9%	6.8%	5,817
CVD mortality	80.2%	4.3%	10.3%	5.2%	1,090
Cancer mortality	79.4%	4.3%	9.6%	6.7%	2,950
Other mortality	75.5%	6.5%	10.2%	7.8%	1,777

NSSEC – National Statistics socio-economic classification
PMV – private motorised vehicle
Source: Office for National Statistics (ONS) Longitudinal Study of England and Wales

Table 15: Cox regression analyses of change in commute mode between1991 and 2001 (active and public versus private motorised vehicle) and mortality and cancer outcomes from the ONS Longitudinal Study 2001-2015/16

Private motorised vehicle users in 1991s

	**All-cause mortality**		**CVD mortality**		**Cancer incidence**		**Cancer mortality**	
**Commute mode**	**Unadjusted HR (95%CI)**	**Adjusted HR (95%CI)**	**Unadjusted HR (95%CI)**	**Adjusted HR (95%CI)**	**Unadjusted HR (95%CI)**	**Adjusted HR (95%CI)**	**Unadjusted HR (95%CI)**	**Adjusted HR (95%CI)**
Continuing private motorised vehicle user	1.00	1.00	1.00	1.00	1.00	1.00	1.00	1.00
Switched from private motorised vehicle to active or public transport	0.97 (0.89, 1.06)	0.93 (0.84, 1.02)	0.92 (0.75, 1.13)	0.96 (0.77, 1.19)	0.90 (0.84, 0.97)	0.97 (0.90, 1.04)	0.88 (0.77, 1.00)	0.88 (0.77, 1.01)
	Failures = 5098	N = 85451	Failures = 972	N = 85451	Failures = 8333	N = 85451	Failures = 2592	N = 85451

Active/public transport users in 1991

	**All-cause mortality**		**CVD mortality**		**Cancer incidence**		**Cancer mortality**	
**Commute mode**	**Unadjusted HR (95%CI)**	**Adjusted HR (95%CI)**	**Unadjusted HR (95%CI)**	**Adjusted HR (95%CI)**	**Unadjusted HR (95%CI)**	**Adjusted HR (95%CI)**	**Unadjusted HR (95%CI)**	**Adjusted HR (95%CI)**
Continuing active or public transport user	1.00	1.00	1.00	1.00	1.00	1.00	1.00	1.00
Switched from active or public transport to private motorised vehicle	0.64 (0.59, 0.71)	0.99 (0.89, 1.09)	0.61 (0.49, 0.76)	0.93 (0.74, 1.19)	0.77 (0.72, 0.83)	1.05 (0.97, 1.14)	0.66 (0.58, 0.76)	0.97 (0.84, 1.13)
	Failures = 1847	N = 31836	Failures = 330	N = 31836	Failures = 2736	N = 31836	Failures = 876	N = 31836

*Adjusted for: age at baseline; age at baseline squared; sex; ethnicity (minority ethnic group, white); housing tenure (homeowner at both, homeowner to renting, renting at both, renting to homeowner); marital status (unmarried at both, unmarried to married, married at both, married to unmarried); education (has a university degree at 1991 or 2001); access to a car (no access at both, no access to access, access at both, access to no access); presence of a long term illness (not ill at both, not ill to ill, ill at both, ill to not ill), National Statistics Social-economic Classification at baseline (higher, intermediate, routine/manual); Change in National Statistics Social-economic Classification (unchanged NSSEC, increased NSSEC, decreased NSSEC); individual level Carstairs quintile at baseline (a composite of male unemployment, lack of car ownership, overcrowding and social class of household head); change in Carstairs quintile (unchanged deprivation quintile, moved to higher deprivation quintile, moved to a lower deprivation quintile); population density (<2000 persons per km^2 in both, moves from <2000 to 2000+ persons per km^2, 2000+ persons per km2 in both, from 2000+ to <2000 persons per km^2)

PMV – private motorised vehicle

Source: Office for National Statistics (ONS) Longitudinal Study of England and Wales

Table 16: Cox regression analyses of commute mode and mortality using the ONS Longitudinal Study 2001-2011 with and without adjustment for self-reported health

	**All-cause mortality**		**CVD mortality**	
**Commute mode**	**HR (95%CI)**	**Maximally Adjusted HR (95%CI)**	**HR (95%CI)**	**Maximally Adjusted HR (95%CI)**
Private motorised vehicle	1.00	1.00	1.00	1.00
Public Transport	0.92 (0.84, 1.01)	0.93 (0.85, 1.02)	0.88 (0.71, 1.09)	0.89 (0.71, 1.10)
Walk	1.02 (0.93, 1.12)	1.02 (0.93, 1.12)	0.99 (0.79, 1.22)	0.99 (0.80, 1.23)
Cycle	0.80 (0.68, 0.96)	0.81 (0.68, 0.97)	0.68 (0.46, 1.03)	0.69 (0.46, 1.03)
Age at baseline	1.14 (1.12, 1.15)	1.13 (1.11, 1.15)	1.19 (1.15, 1.23)	1.19 (1.14, 1.23)
Age at baseline squared	1.00 (1.00, 1.00)	1.00 (1.00, 1.00)	1.00 (1.00, 1.00)	1.00 (1.00, 1.00)
Women	0.60 (0.56, 0.64)	0.60 (0.56, 0.64)	0.32 (0.27, 0.37)	0.32 (0.27, 0.37)
Married	0.79 (0.74, 0.84)	0.79 (0.74, 0.85)	0.73 (0.64, 0.84)	0.74 (0.64, 0.85)
Black minority ethnic	0.72 (0.63, 0.83)	0.72 (0.63, 0.83)	0.91 (0.69, 1.21)	0.91 (0.68, 1.21)
Has access to a car	0.61 (0.56, 0.68)	0.62 (0.57, 0.69)	0.64 (0.52, 0.79)	0.65 (0.52, 0.80)
Population density 2000+ persons/km^2	1.01 (0.95, 1.08)	1.01 (0.95, 1.08)	0.94 (0.82, 1.08)	0.94 (0.82, 1.08)
Entered study 1991	1.00	1.00	1.00	1.00
Entered study 2001	0.44 (0.41, 0.47)	0.44 (0.41, 0.47)	0.33 (0.28, 0.39)	0.33 (0.28, 0.39)
Has a degree	0.80 (0.74, 0.88)	0.81 (0.74, 0.88)	0.61 (0.49, 0.75)	0.61 (0.49, 0.76)
Non-home owner	1.25 (1.16, 1.35)	1.23 (1.14, 1.33)	1.38 (1.17, 1.62)	1.36 (1.15, 1.60)
**Socio-economic classification (NSSEC)**				
Higher	1.00	1.00	1.00	1.00
Intermediate	1.04 (0.95, 1.13)	1.04 (0.95, 1.13)	1.10 (0.92, 1.33)	1.10 (0.92, 1.33)
Routine/Manual	1.16 (1.07, 1.24)	1.15 (1.07, 1.24)	1.14 (0.97, 1.34)	1.14 (0.97, 1.34)
**Carstairs Deprivation Index Quintile**				
Least deprived	1.00	1.00	1.00	1.00
Next least deprived	1.04 (0.95, 1.13)	1.04 (0.95, 1.13)	1.27 (1.05, 1.55)	1.27 (1.04, 1.54)
Mid-point	1.04 (0.95, 1.13)	1.03 (0.94, 1.12)	1.17 (0.95, 1.43)	1.16 (0.94, 1.42)
Moderate deprivation	1.11 (1.01, 1.22)	1.10 (1.00, 1.21)	1.28 (1.04, 1.59)	1.27 (1.02, 1.57)
Most deprived	1.12 (1.01, 1.24)	1.10 (0.99, 1.22)	1.36 (1.08, 1.72)	1.35 (1.06, 1.70)
Has a long term illness at baseline	1.84 (1.67, 2.04)	1.50 (1.35, 1.66)	2.27 (1.85, 2.79)	1.93 (1.55, 2.40)
Health is not good or fairly good		2.19 (2.00, 2.41)		1.97 (1.61, 2.42)
Failures=4,963 N=209371			Failures = 1000 N=209371	

NSSEC – National Statistics socio-economic classification
Source: Office for National Statistics (ONS) Longitudinal Study of England and Wales

Table 17: Cox regression analyses of commute mode and mortality outcomes using the ONS Longitudinal Study 1991-2016, excluded the first 2 years of follow-up in each 10 year period

	**All-cause mortality**		**CVD mortality**		**Cancer incidence**		**Cancer mortality**	
	**Unadjusted**	**Adjusted**	**Unadjusted**	**Adjusted**	**Unadjusted**	**Adjusted**	**Unadjusted**	**Adjusted**
**Commute mode**	**HR (95%CI)**	**HR (95%CI)**	**HR (95%CI)**	**HR (95%CI)**	**HR (95%CI)**	**HR (95%CI)**	**HR (95%CI)**	**HR (95%CI)**
Private motorised	1.00	1.00	1.00	1.00	1.00	1.00	1.00	1.00
Public transport	1.01 (0.96, 1.06)	0.99 (0.93, 1.05)	0.94 (0.85, 1.05)	0.90 (0.79, 1.02)	0.80 (0.76, 0.83)	0.93 (0.88, 0.98)	0.92 (0.85, 0.99)	0.98 (0.90, 1.07)
Walk	1.19 (1.12, 1.25)	0.99 (0.93, 1.05)	1.07 (0.95, 1.20)	0.92 (0.81, 1.05)	1.00 (0.95, 1.05)	0.92 (0.87, 0.97)	1.17 (1.08, 1.27)	0.99 (0.91, 1.08)
Cycle	0.94 (0.85, 1.05)	0.80 (0.72, 0.90)	0.95 (0.76, 1.19)	0.75 (0.60, 0.95)	0.86 (0.78, 0.94)	0.89 (0.81, 0.98)	0.93 (0.79, 1.08)	0.88 (0.75, 1.03)
	Failures=11,893	N=392996	Failures=2,618	N = 392996	Failures = 17015	N = 390402	Failures=5611	N = 392996

*Adjusted for: age at baseline; age at baseline squared; sex; ethnicity (minority ethnic group, white); housing tenure (homeowner, non-homeowner); marital status (married, non-married); education (no degree, has a degree); access to a car (yes, no); National Statistics Social-economic Classification (higher, intermediate, routine/manual); year entered study and presence of a long term illness at baseline (yes, no), neighbourhood measures included were population density (<2000 persons per km2, 2000+ persons per km^2) and individual level Carstairs quintile (a composite of male unemployment, lack of car ownership, overcrowding and social class of household head)

Source: Office for National Statistics (ONS) Longitudinal Study of England and Wales

Table 18: Cross tabulation of commute modes for this present in consecutive censuses

1991 and 2001

	**Commute mode 2001**										
	Private motorised		Bus		Rail		Walking		Cycling		Total
**Commute mode 1991**											
Private motorised	76,071	89%	2,050	2%	2,123	2%	3,667	4%	1,436	2%	85,347
Bus	5,509	52%	3,036	29%	599	6%	1,157	11%	242	2%	10,543
Rail	2,912	39%	466	6%	3,572	48%	315	4%	117	2%	7,382
Walking	6,467	48%	1,167	9%	370	3%	4,890	37%	452	3%	13,346
Cycling	2,162	52%	180	4%	110	3%	408	10%	1,260	31%	4,120

2001 and 2011

	**Commute mode 2011**										
	Private motorised		Bus		Rail		Walking		Cycling		Total
**Commute mode 2001**											
Private motorised	85,589	88%	2,117	2%	3,145	3%	4,555	5%	1,525	2%	96,931
Bus	4,340	47%	2,783	30%	729	8%	1,119	12%	194	2%	9,165
Rail	3,002	33%	522	6%	4,740	53%	461	5%	242	3%	8,967
Walking	5,747	47%	950	8%	479	4%	4,723	38%	397	3%	12,296
Cycling	2,016	50%	160	4%	155	4%	410	10%	1,267	32%	4,008

Source: Office for National Statistics (ONS) Longitudinal Study of England and Wales
